# Exploration of the prognostic signature reflecting tumor microenvironment of lung adenocarcinoma based on immunologically relevant genes

**DOI:** 10.1080/21655979.2021.1974779

**Published:** 2021-10-06

**Authors:** Wei Wu, Liye Jia, Yanan Zhang, Juanjuan Zhao, Yunyun Dong, Yan Qiang

**Affiliations:** a Department of Physiology, Shanxi Medical University, Taiyuan, China; b Key Laboratory of Cellular Physiology, (Shanxi Medical University), Ministry of Education, Taiyuan, China; c Key Laboratory of Cellular Physiology, Shanxi Province, Taiyuan, China; d College of Information and Computer, Taiyuan University of Technology, Taiyuan,China; e School of Software, Taiyuan University of Technology, Taiyuan, China

**Keywords:** Lung adenocarcinoma, immunologically relevant gene, tumor microenvironment, microenvironment, prognosis, signature, RiskScore

## Abstract

Lung adenocarcinoma (LUAD) represents the major histological type of lung cancer with high mortality globally. Due to the heterogeneous nature, the same treatment strategy to various patients may result in different therapeutic responses. Hence, we aimed to elaborate an effective signature for predicting patient survival outcomes. The TCGA-LUAD cohort from the TCGA portal was used as a training dataset. The GSE26939 and GSE68465 cohorts from the GEO database were taken as validation datasets. All immunologically relevant genes were extracted from the ImmPort. The ESTIMATE algorithm was employed to explore LUAD microenvironment in the training dataset. Further, the DEGs were picked out based on the immune-associated genes reflecting different statuses in the immune context of TME. Univariate/multivariate Cox regression was performed to determine six prognosis- specific genes (PIK3CG, BTK, VEGFD, INHA, INSL4, and PTPRC) and established a risk predictive signature. The time-dependent ROC indicated that AUC values were all greater than 0.70 at 1-, 3-, and 5- year intervals. Corresponding RiskScore of each LUAD patient was calculated from the signature, and they were stratified into the high- and low-risk groups by the median value of RiskScore. K-M curves and Log-rank test demonstrated significant survival differences between the two groups (*P* < 0.05). Similar results were exhibited in the validation datasets. The RiskScore was incredibly relevant to clinicopathological factors like gender, AJCC stage, and T stage. Also, it can mirror the distribution state of 15 kinds of TIICs and have some predictive value for the sensitivity of therapeutic drugs.

## Introduction

Lung cancer is the second leading aggressive malignancy after breast cancer, responsible for 18% of all tumor-related deaths [1,2]. Lung adenocarcinoma (LUAD) is the primary histopathological type of lung cancer [3]. Since there are insidious and symptomless at the early stage, it is commonly diagnosed in advanced stages with local invasion or distant metastasis [4]. Therefore, this contributes to missing the therapeutic opportunities to gain surgical procedures and even causes a poor prognosis. The advent of immunotherapy has brought a unique perspective to pave the way for advanced LUAD patients. More recently, the immunotherapy targeting immune checkpoint

molecules like programmed cell death protein 1 receptor/ ligand (PD-1, PD-L1) and cytotoxic T lymphocyte antigen-4 (CTLA-4) has yielded encouraging outcomes [5–7], emphasizing the essential role of the tumor microenvironment (TME) to influence the clinical therapeutic outcome. Nowadays, it is generally accepted that immunotherapy is to recruit the immune cells within or outside the TME to recognize and kill the cancer cells by targeting tumor surface differentiation antigens, which can induce a protective antitumor response to suppress immune escape of neoplastic cells [8]. However, the efficacy of this method is nearly 15–20% [9]. Furthermore, it might produce a series of immune-related adverse events and acquire drug resistance [10,11]. At the same time, the cost of immunotherapy is enormous [12]. Accordingly, it is imperative to identify the beneficiary population and predict patient survival from the immunotherapy.

With significant advances in high-throughput sequencing technology combined with bioinformatics analytic methods, more novel prognostic and predictive biomarkers of LUAD have been mined. The signature consisting of 30 immune-related genes could be recognized as an independent factor for the survival time of LUAD patients [13]. SPRR1B gene was identified as a prognosis predictor in LUAD via being screened by bioinformatics analysis methods and then verified using cell biology experiments [14]. In particular, increasing attention has been focused on the relationship between TME and the projection of LUAD. Four immune-related prognostic biomarkers were screened by integrating five public microarray datasets, and the expression level of markers was positively correlated with different types of tumor-infiltrating immune cells [15]. Chen et al. [16] have revealed TME-based signatures capable of predicting LUAD patient survival and therapeutic responses. However, large amounts of studies tended to mainly concentrate on a single critical gene/pathway or one aspect of tumor development. They paid little attention to tumor crosstalk among different driving factors. Therefore, it is necessary to search for the broader signatures from the view of multiple factors that could accurately predict the clinical efficacy of varying immunotherapy approaches and prognostic information for LUAD patients. In this present study, we hypothesized that continuous crosstalk between immunologically relevant genes and the immune cell infiltration in the TME could provide clues for predicting survival outcome and immunotherapy response with LUAD patients. The goal of our present study was to uncover appropriate prognostic signatures from both immune-associated gene profiles and the surrounding microenvironment of LUAD cells aspects. Specifically, the immune-associated genes were obtained from the ImmPort database. The microenvironment of LUAD was analyzed by means of the ESTIMATE algorithm, and further a list of TME-specific genes was extracted. The immune-TME-related genes as candidate biomarkers were identified from the above two gene sets. As a result, we innovatively proposed a prognostic signature of six genes through univariable/multivariate Cox regression analysis. Besides, both internal and external validation was carried out to verify the effectiveness of this prediction signature. Finally, it is expected that these results are to provide a more comprehensive picture of the immunogenomic landscape of TME and a better prognosis predictor for patient stratification and personalized precision treatment.

## Materials and methods

### Data collection of TCGA & GEO databases

Raw transcriptome RNA-sequencing (RNA-seq) and clinical pathology information of The Cancer Genome Atlas (TCGA) LUAD patients were obtained from the Genomic Data Commons (GDC) data portal (https://portal.gdc.cancer.gov/). The GSE26939 and GSE68465, including the mRNA expression and matching clinical data of patients with LUAD, were retrieved from the database of the National Center for Biotechnology Information (NCBI) Gene Expression Omnibus (GEO) (https://www.ncbi.nlm.nih.gov/geo/). The GSE26939 dataset was analyzed using the GPL9053 Agilent-UNC-custom-4X44K microarray chip platform, submitted by Wilkerson et al. [17]. The GSE68465 was described based on the [HG-U133A] Affymetrix Human Genome U133A Array (GPL96) platform by Shedden et al. [18]. The data of TCGA-LUAD was used as a training dataset for establishing the risk prognostic signature, whereas data in GSE26939 and GSE68465 were applied to verify the effectiveness of the signature.

### Analysis of TME components in TCGA-LUAD dataset

To evaluate the cellular heterogeneity of the TME for each LUAD patient, it was necessary to quantify the TME using the cancer tissue genomic and transcriptomic spectrums. ESTIMATE algorithm was exploited to analyze tumor purity and non- neoplastic cell infiltration based on ssGSEA [19]. The R package ‘ESTIMATE’ was implemented to RNA-seq transcriptome profiles to calculate three types of score in tumor tissue: ImmuneScore that quantifies the abundance of infiltrating immune cells, StromalScore that denotes the presence of stromal cells, and ESTIMATEScore that is the sum of the above ImmuneScore and StromalScore which re presenting the composite proportion of these two components in TME. Then, Kaplan-Meier (K-M) survival analyses and Log-rank test were constructed for ImmuneScore, StromalScore, and ESTIMATEScore, respectively. Associations between three scores and clinical parameters were assessed by the Kruskal-Wallis test or the Wilcoxon test. Differences were taken to be statistical significance if *P* < 0.05.

### Generation of differentially expressed genes (DEGs)

First, all TCGA-LUAD patients were classified into high vs. low score subgroups by the median ImmuneScore/StromalScore split, respectively. Genes that were upregulated (downregulated) among high ImmuneScore and StromalScore subgroups were taken as co-upregulated (co-downregulated) DEGs. Only genes that commonly appeared in these two intersection sets were considered as the significant TME-specific DEGs. DEGs were picked out using the ‘limma’ R package [20]. The selection criteria were |log2(fold change) | > 1 and false discovery rate (FDR) < 0.05. And heatmaps and clustering of DEGs were visualized by the R package ‘pheatmap’ (https://CRAN.R- project.org/package = pheatmap). Then, the entire set of immune-associated genes was downloaded from ImmPort (https://www.ImmPort.org/) [21], which is the NIH-funded bioinformatics repository for the field of immunology. Finally, given that the two categories of gene-sets identified above, the overlapping genes were further selected as the immune-TME-related DEGs. In parallel, the DEGs among different groups were depicted with the R library ‘VennDiagram’ (https://CRAN.R- project.org/package = VennDiagram).

### Identification of the risk prognostic signature

To determine the association between immune-TME-related DEGs and prognostic, the univariate Cox proportional hazards regression analysis was employed. And genes, which were found to be considered significant with a cutoff of *P* < 0.01, were suggested as candidates. Then, the weight of each gene was also generated via a stepwise multivariate Cox regression model. All analyses were executed by the function of coxph in the R ‘survival’ package (https://cran.r-project.org/package = survival). Moreover, the risk prognostic signature was constructed through the sum of each gene’s expression value multiplied with its respective Cox regression coefficient.

### Evaluation of the risk prognostic signature in TCGA-LUAD dataset

The RiskScore of each LUAD individual was calculated by the risk prognostic formula.

The RiskScore distribution curve, survival status scatter plot, and heatmap were drawn by the R language tool. All LUAD patients were split into a high-risk group and a low- risk group by the median RiskScore. Differences in the clinicopathological parameters between the two groups, as mentioned above, were compared via Chi-square tests. Heatmaps were generated using the ‘ComplexHeatmap’ package in R environment [22]. K-M survival curves coupled with Log-rank test were performed using the R packages ‘survival’ and ‘survminer’ (https://CRAN.R- project.org/package = survminer). The specificity and sensitivity of the prognosis signature were assessed by time-dependent receiver operating characteristic (ROC) at 1-, 3- and 5-years using the R package ‘timeROC’ [23]. Area under the curve (AUC) values were calculated. Additionally, in order to determine whether the RiskScore and clinical indices (such as age, gender, AJCC stage, and TNM stage) were independent predictors of LUAD prognostic, the proportional hazard model of Cox regression was conducted.

### Validation of the performance of the risk prognostic signature in the GEO repository

The datasets from the GEO were externally validated, accession number GSE26939 and GSE68465. The RiskScore specific to per LUAD patient was computed using the risk equation from the training dataset. The median RiskScore was a useful threshold for categorizing all patients as high- and low-risk groups. K-M plotter (Log-rank test) and time-dependent ROC curve were carried out for further verifying the generalization of the prognostic signature with R program (Version: 4.0.3).

### Relationship between riskscore and the 22 kinds of tumor-infiltrating Immune cells (TIICs)

To identify whether the RiskScore could reflect the state of TME in cancerous tissue, we further analyzed the relation of 22 TIICs with the RiskScore in the TCGA-LUAD dataset. First, the CIBERSORT deconvolution algorithm was utilized to determine the relative quantity of 22 TIICs with the whole RNA-seq expression profiles [24]. The results were mapped to the histogram and correlation matrix visually with the ‘corrplot’ package on R software [25]. Second, the degree of TIICs infiltration was compared between risk groups by the Wilcoxon test. Violin plots were applied to show the distribution of the difference in diverse types of TIICs. Finally, the Spearman correlation test was implemented for estimating the association between the RiskScore and tumor-infiltrating immune components. When *P* values were both less than 0.05 in differences and correlations analyses, TIICs were thought to be statistically significant and considerably associated with the RiskScore.

### Assessment of the sensitivity of therapeutic agents

The sensitivity of multiple drugs among different risk groups was evaluated by 50% inhibitory concentration (IC50). The IC50 of each drug was computed using ridge regression from the Genomics of Drug Sensitivity in Cancer (GDSC, https://www.cancerrxgene.org) pharmacogenomics database. Data analyses were done with the package ‘pRRophetic’ in R [26]. The selected drugs, including cisplatin, paclitaxel, gemcitabine, and vinorelbine, are the first-line medication for the treatment of lung cancer in clinical practice. Apart from that, the immunotherapeutic effect of blockade antibodies targeted PD1 and CTLA4 was also assessed by the Tumor Immune Dysfunction and Exclusion (TIDE) prediction score (http://tide.dfci.harvard.edu) [27,28]. The results in the form of box plots were charted by R package ‘ggplot2’ (https://ggplot2.tidyverse.org/) [29]. Differences were indicated significant at *P*-value < 0.05.

## Results

In this study, we hypothesized that immunologically relevant genes reflecting the status of TME could provide leads for predicting survival and immunotherapy response with LUAD patients. Therefore, we innovatively proposed an immune-TME-related signature that can not only stratify the risk of LUAD patients but also aid in clinical decision-making. Firstly, the ESTIMATE algorithm and immune-associated genes from the ImmPort database were employed to identify candidate biomarkers. Secondly, a risk signature based on targeted markers was established and verified the predictive performance in terms of prognosis and drug sensitivity. It analyzed the correlation with immune cell infiltration in TME. Finally, the effectiveness of this signature was validated in the two additional datasets, GSE26939 and GSE68465.

### The landscape of TME based on immunescore, stromalscore, and ESTIMATEScore

**Figure 1.**
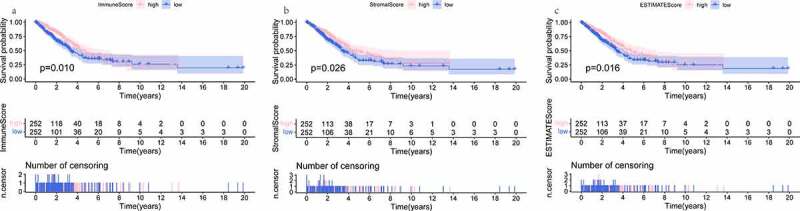
K-M analyses showing the predictive capability of three types of scores with the prognosis of LUAD patients. (a) comparison of OS rate in high versus low ImmuneScore groups. (b) comparison of OS rate in high versus low StromalScore groups. (c) comparison of OS rate in high versus low ESTIMATEScore groups

**Figure 2.**
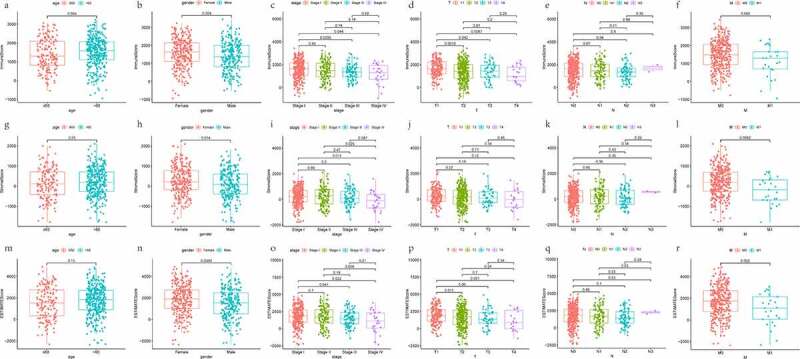
Scatter plots represent the association between the three scores of TME and clinical indicators (age, gender, AJCC stage, T stage, N stage, and M stage). (a-f) The distribution of ImmuneScore. (g-l) the distribution of StromalScore. (m-r) the distribution of ESTIMATEScore

The distributions of demographic and clinical information for all datasets were presented in Table 1. TCGA-LUAD dataset contained 515 cases. A total of 594 transcriptome sequencing data were obtained, 535 (90.1%) came from tumor samples and 59 (9.9%) were derived from normal samples. First, the infiltration levels of immune cells, the content of stromal cells, and the purity of tumor cells for each tumor sample were scored by the ESTIMATE computational method according to FPKM data of mRNA. And the population was dichotomized at the median scores, respectively. Then, the comparison of overall survival (OS) was performed by K-M analysis and Log-rank test. As shown in Figure 1, the patients in the high ImmuneScore/StromalScore subgroup had more favorable outcomes than those in the respective low-score subgroup (both *P* < 0.05). The high ESTIMATEScore group, which meant the low content of tumor cells, show ed a positive correlation with the OS rate. Finally, to investigate whether the proportion of immune/stromal components was associated with clinical parameters, we investigated the correlation among them. The results shown in Figure 2, ImmuneScore was related to patient gender, AJCC stage, T stage, and N stage. Remarkably, regarding the T stage, a higher level of ImmuneScore was found in patients with T1 stage compared to T2, T3, and T4 stage, respectively (all *P* < 0.05). These suggested that the infiltration extent of immune cells in the early phase of LUAD was significantly greater than that in other phases. StromalScore was correlated with gender, AJCC stage, and M stage. Notably, compared with the M0 stage, StromalScore in the M1 stage was significantly decreased (*P* < 0.05). As it turned out, the tumor tissues with distant metastasis contained fewer stromal cells.

**Table 1.** Summary of patients’ demographic and clinical characteristics in all datasets

Features	Training dataset	Validation dataset	
	TCGA-LUAD n = 515	GSE26939 n = 116	GSE68465 n = 462
Age at diagnosis, years			
≤ 60	159	40	147
> 60	337	76	296
Unknown	19	NA	19
Gender			
Female	276	63	220
Male	239	53	223
Unknown	0	0	19
AJCC stage			
I	276	50	NA
II	121	16	NA
III	84	17	NA
IV	26	1	NA
Unknown	8	32	NA
T stage			
T1	169	NA	150
T2	277	NA	251
T3	47	NA	28
T4	19	NA	12
Unknown	3	NA	21
N stage			
N0	332	NA	299
N1	95	NA	88
N2	74	NA	53
N3	2	NA	0
Unknown	12	NA	22
M stage			
M0	346	NA	NA
M1	25	NA	NA
Unknown	144	NA	NA
Survival status			
Alive	328	49	207
Dead	187	66	236
Unknown	NA	1	19

ESTIMATEScore was related to gender, AJCC stage, T stage, and M stage. Clinicopathologic variables, including gender, primary tumor, and distant metastasis, might affect the tumor purity of LUAD. Taken together, the presence of immune and stromal cell populations in TME plays a fundamental role during LUAD development, invasion, and metastasis.

### Identification of the differentially expressed immunologically relevant genes that reflecting the LUAD microenvironment

**Figure 3.**
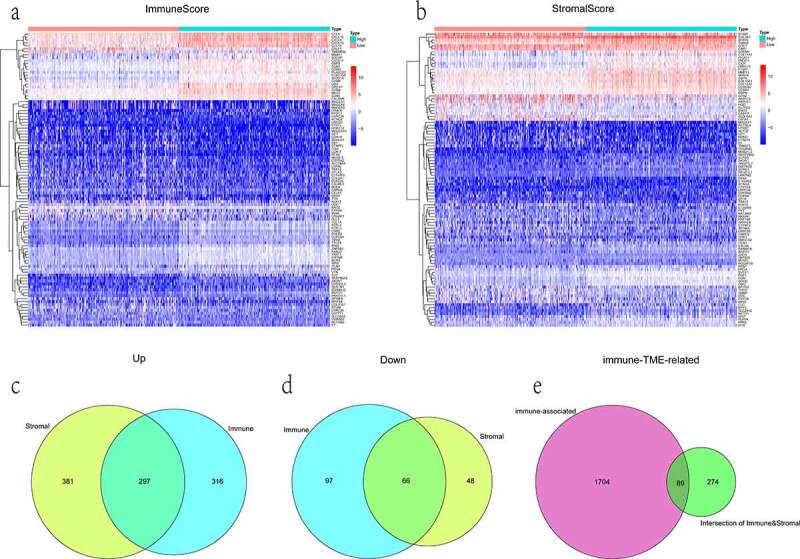
The display of DEGs among different groups. (a, b) heatmaps revealed DEGs by comparing high- and low-score groups in ImmuneScore and StromalScore, respectively. The abscissa represents the ID of patient samples, and the ordinate represents the name of DEGs. The top 50 DEGs were listed. (c, d) venn diagrams were depicted for upregulated and downregulated DEGs based on shared genes in ImmuneScore and StromalScore groups, respectively. (e) venn plot showed the overlap of immunologically relevant genes and TME-specific genes

A number of studies have shown that immune-associated genes might reflect the status of the TME, regulating tumor progression and maintenance [30,31]. A total of 2,483 immunologically relevant genes were downloaded. Among them, duplicate genes were deleted, leaving 1,793 unique genes. To identify TME-specific DEGs, differential expression analysis was conducted. In comparing high and low ImmuneScore groups, 613 genes were found to be upregulated in the high-score group, while 163 genes were downregulated. And in the comparison based on StromalScore, 678 upregulated and 114 downregulated genes were detected as well. Genes corresponding to the mentioned categories for both groups were displayed in the form of heatmaps (Figure 3(a-b)).

Moreover, the Venn diagram showed the intersectional analyses of sharing DEGs in ImmuneScore and StromalScore groups. Results were presented that 363 genes were filtered, in which 297 were upregulated and 66 were downregulated (Figure 3(c-d)). Subsequently, the 89 overlap genes between immune-associated and TME-specific were picked out as immune-TME-related DEGs for the following analysis (Figure 3(e)).

### Construction of the risk predictive signature for the prognosis of LUAD patient

According to the previous findings, 9 out of 89 DEGs were highly related to survival status by univariate Cox proportional hazards regression analysis. Among these 9 genes, 6 genes with maximum prognostic value, including 3 beneficial (coefficient < 0) and 3 risky (coefficient > 0) genes, were further screened out using multivariate Cox analysis (Table 2). 6 genes were incorporated into the prognostic signature as variables. The RiskScore of each LUAD sample was computed referring to the following formula: RiskScore = (−0.325 × ExpPIK3CG) + (−0.102 × ExpBTK) + (−0.095 × ExpVEGFD) + (0.005 × ExpINHA) + (0.010 × ExpINSL4) + (0.050× ExpPTPRC).

**Table 2.** The parameters for establishing the multivariate Cox proportional hazards regression signature

ID	Coef	HR	HR.95%L	HR.95%H	*P* value
PIK3CG	−0.324579	0.722831	0.532872	0.980507	0.036933
BTK	−0.102129	0.902913	0.788235	1.034274	0.140567
VEGFD	−0.094507	0.909821	0.848513	0.975559	0.007927
INHA	0.004911	1.004923	0.999287	1.010591	0.086996
INSL4	0.010271	1.010323	1.000381	1.020365	0.041807
PTPRC	0.050434	1.051728	0.995622	1.110996	0.071374

Coef: Cox coefficient; HR: hazard ratio.

### Analysis of the predictive efficacy of the prognostic signature

**Figure 4.**
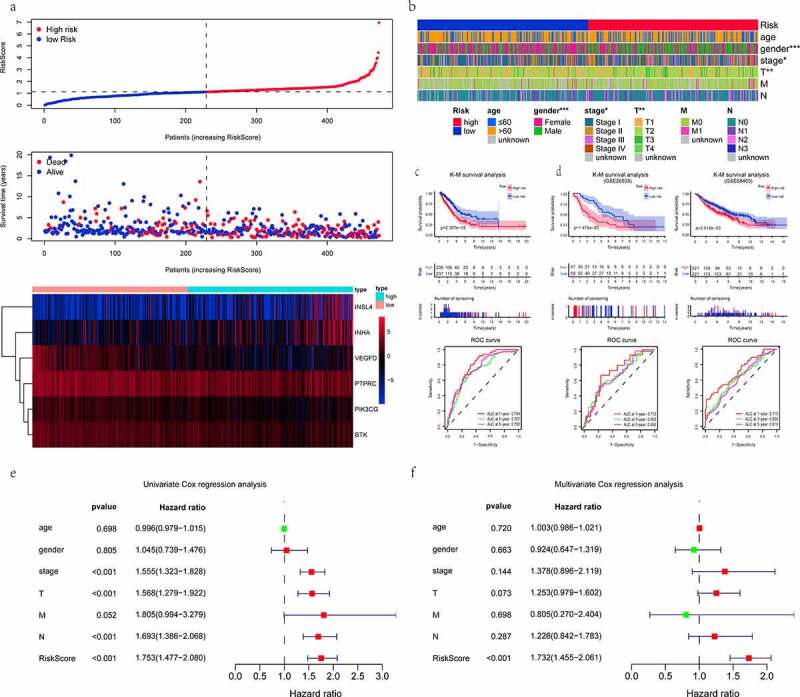
Evaluation and validation of the risk prognostic signature. (a) RiskScore distribution, survival overview, and heatmap for patients in the TCGA-LUAD dataset assigned to high- and low-risk groups based on the RiskScore median. (b) heatmap of the correlation between the two groups and clinicopathological parameters. *: *P* < 0.05, **: *P* < 0.01, ***: *P* < 0.001. (c, d) K-M survival and time-dependent ROC analyses were performed to predict the prognosis and the efficiency of the risk signature for high-/low-risk groups in training and validation datasets, respectively. (e, f) univariate/multivariate Cox regression analyses showing the independent prognostic value of RiskScore in TCGA-LUAD

The median value of RiskScore calculated by the above equation was taken as a cutoff value. Of the 473 patients, 236 were in the high-risk group and 237 were in the low- risk group. The distribution of the RiskScore, survival status and expression patterns of 6 genes were revealed in Figure 4(a). As can be seen from the thermogram of relatedness (Figure 4(b)), the group with a low risk had a significantly greater proportion of female patients as compared to the group with a high risk (*P* < 0.001). And there were more patients in the low-risk group with AJCC stage I (*P* < 0.05) and T1 stage (*P* < 0.01), while those of the high-risk were in the middle-advanced stage. It further illustrated the major of low-risk patients were at an early stage of tumor progression. RiskScore was helpful to stratify patients with high or low risk. K-M survival analysis (Log-rank test) obviously showed that, unlike the low-risk group, the high-risk patients were associated with a worse prognosis (*P* < 0.001). The 5-year survival rate was 32.6% (95% confidence interval [CI] = [24.9–42.6%]) for the high-risk group versus 49.7% (95% CI = [39.6–62.4%]) for the low-risk group. In addition, the predictive power of this risk prognostic signature was measured by the time-dependent ROC. The AUC values of 0.794, 0.727, and 0.760 were displayed at the 1-, 3-, and 5-year, respectively (Figure 4(c)). Next, we examined that the RiskScore could view as an independent prognostic indicator for LUAD patients irrespective of other clinical characteristics using the Cox proportional hazards model. The HR was roughly 1.7 (*P* < 0.001). More specifically, univariate Cox analysis implied that the clinical factors (tumor AJCC stage, T, and N stage) were also associated with the prognosis of LUAD (Figure 4(e-f)). Collectively, the RiskScore had the greatest influence on predicting the survival rate, indicating that the six-gene-based risk signature could better predict the LUAD patient’s prognostic status.

### The validation of the prognostic signature in the external dataset

To ensure the feasibility and accuracy of this signature, it was applied to the GEO database. GSE26939 dataset contains 116 patients with LUAD, but one patient (GSM663361) was excluded due to missing survival information. The GSE68465 dataset comprises a total of 462 samples, where 19 samples are normal and the rest are LUAD tumor samples. One LUAD patient (GSM1672389) was also ruled out because of the same reason as described above. Subsequently, the K-M analysis curve (Log- rank test) implied that survival time was significantly shorter in the high-risk group than in the low-risk group (both *P* < 0.01). The time-dependent ROC analysis results revealed that the AUC at 1-, 3- and 5-year OS of the GSE26939 and GSE68465 were 0.710, 0.663, 0.665, 0.719, 0.630, 0.618, respectively (Figure 4(d)). In general, these results further illustrated that the prognostic signature based on immune-TME-related DEGs was robust in predicting the survival rates of patients with LUAD.

### Correlation between the RiskScore and the proportion of TIICs

**Figure 5.**
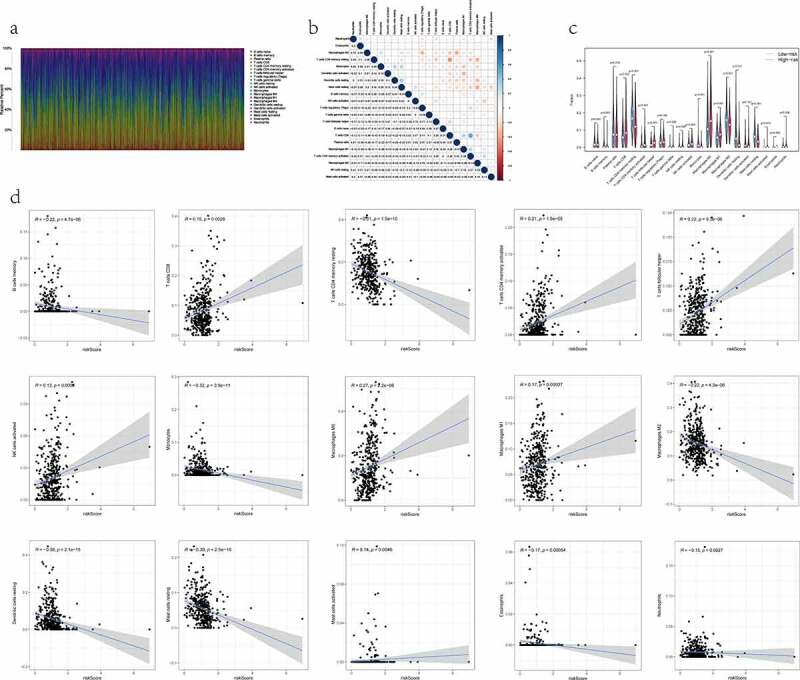
The relationship between the RiskS of our prognostic signature and the proportion of TIICs in the training dataset. (a) histograms showing 21 kinds of TIICs profile for each LUAD patients. The lateral axis represents sample ID and the longitudinal axis represents the relative percent of different types of TIICs. (b) correlation matrix displaying the pairwise correlation between 21 kinds of TIICs. Color or number in each cell depicting the corresponding correlation and *P*-value between two kinds of TIICs, respectively. (c) violin plots visualizing the infiltration fractions of 21 kinds of TIICs between high- and low-risk groups. The horizontal and vertical coordinates represent the name and relative content of TIICs, respectively. (d) the correlation between the RiskScore and significantly different 15 types of TIICs

We quantified the relative composition of multiple immune cell subpopulations infiltrating the TME by CIBERSORT and drew 22 kinds of TIICs profiles from the RNA-seq data of each patient (Figure 5(a)). The results from the analysis showed there were no infiltration fraction of T cells CD4 naïve in all samples. So, we removed this cell type, leaving 21 TIICs for follow-up evaluation. Meanwhile, a heatmap of the correlation matrix between 21 types of TIICs was also displayed (Figure 5(b)). By combining difference and correlation analyses (Figure 5(c-d)), a total of 15 TIICs were tightly linked to the RiskScore. Among them, 7 kinds of TIICs, including mast cells activated, macrophages M1, macrophages M0, T cells follicular helper, T cells CD8, T cells CD4 memory activated, and NK cells activated, were positively correlated with RiskScore. In contrast, the remaining 8 kinds of TIICs, including macrophages M2, mast cells resting, dendritic cells resting, T cells CD4 memory resting, B cells memory, monocytes, neutrophils, and eosinophils, were negatively correlated with RiskScore. Hence, it demonstrated that the RiskScore might reflect the immune cell infiltration status of the TME in LUAD tissue.

### The treatment response of different drugs

**Figure 6.**
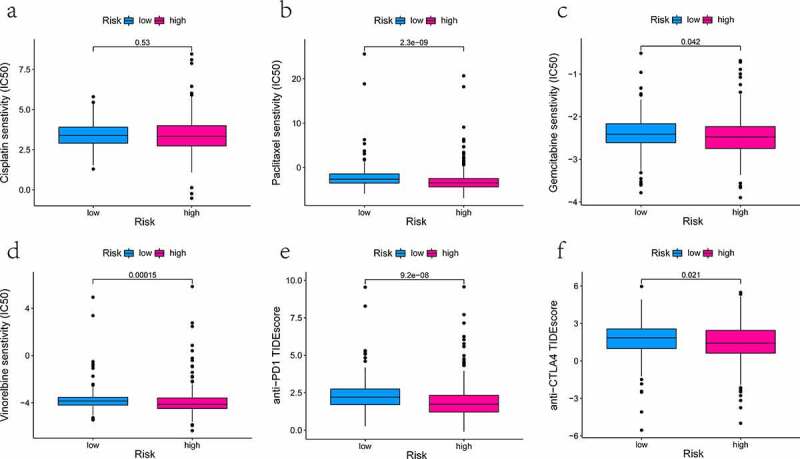
The effectiveness analysis of different types of medication at high- and low-risk group in TCGA-LUAD patients

We have calculated the value of IC50 of four therapeutic drugs in all TCGA-LUAD patients using the pRRophetic algorithm and investigated between-group differences. The observed difference in the IC50 value of cisplatin did not reach statistical significance (Figure 6(a), *P* = 0.53). Then, three drugs (paclitaxel, gemcitabine, and vinorelbine) had lower IC50 for high-risk patients. It hinted that the high-risk group was more susceptible to the above-mentioned drugs (all *P* < 0.05, Figure 6(b-d)). Also, the TIDE score could predict the effectiveness of anti-PD1 and anti-CTLA4 in immunotherapy. Quantifications were presented below in box plots. It was found that patients from the low-risk group had higher TIDE scores compared to those of the high- risk group (*P* < 0.05, Figure 6(e-f)). It was shown that high-risk patients were likely to profit more from anti-PD1/CTLA4 with the lower potential of tumor immune dysfunction and immune escape.

## Discussion

At present, it is strikingly challenging to tailor treatment schemes for malignancies owing to tumor heterogeneity and tumorigenesis mechanisms involving complicated genetic backgrounds. Particularly, in the era of personalized medicine, accurate prognostic assessment is greatly warranted for the clinical treatment and management of LUAD patients. On the one hand, a dynamic interaction between the tumor cells and other cells or extracellular components of its microenvironment could influence the balance in tumor proliferation and suppression. More recently, mounting evidence has indicated that the biological characteristics of TME are intimately linked to the prognosis of many types of cancer [32–34]. Studies about prognostic markers of LUAD microenvironment, Mo et al. found that the hypoxia-associated gene signature could independently deem as a potential prognostic predictor [35]. Zhang et al. reported that a novel model based on eight metabolism-related genes had the more vital predictive ability for LUAD diagnosis and prognosis [36]. Besides, the vascular proliferation index (VPI), which was the ratio between proliferating vessel density and the overall microvessel density, had been established to be a significant prognostic marker [37].

On the other hand, cancer immunoediting could cause the dysregulation of the body’s immune system to result in the eventual escape of tumor cells from the host immune surveillance [38]. Therefore, we speculated that immunologically relevant genes that reflect the status of TME might gain a crucial role in the LUAD progression and prognosis.

The principal objective of our research was to explore and validate promising immune molecular characteristics that reflected the state of TME for LUAD patient’s risk stratification. We engineered a novel prediction signature based on specific candidate genes using the publicly TCGA-LUAD high-throughput transcriptomic data as the training dataset and the microarray data of GSE26939 and GSE68465 from GEO as independent external validation datasets. First of all, all the immune genes were downloaded from the ImmPort database. Second, from the view of immune/stromal cell scores, the TME was quantified by the ESTIMATE algorithm in TCGA-LUAD tumor tissue. Our primary analysis showed that the cellular and the stromal composition of the TME could impact survival and have a great relevance with clinical features in LUAD patients. Afterward, the immune-TME-related DEGs were screened out and constructed a risk appraisal signature according to univariate/multivariate Cox regression. Eventually, taking a diversity of perspectives, the reliability of signature was evaluated by K-M survival (Log-rank test), time-dependent ROC curve, and other analytical methods. The validation datasets of GSE26939 and GSE68465 were in good consistent prediction result with the TCGA-LUAD dataset. The RiskScore calculated the current risk signature was an independent predictor of survival in LUAD patients.

Moreover, patients with high RiskScore indicated worse survival outcomes. Our study’s findings went one step further, suggesting that the RiskScore was closely related with 15 kinds of TIICs by the CIBERSORT algorithm and had the predictive value for the sensitivity and availability of LUAD therapeutic drugs to some extent. In a word, our immune-TME-specific signature may be a useful, practical tool in clinical prognosis prediction and treatment decision-making for LUAD patients.

The predictive signature contained six genes selected by bioinformatical analysis, which were PIK3CG, BTK, VEGFD, INHA, INSL4, and PTPRC, respectively. Among these genes, PIK3CG, BTK, VEGFD were favorable prognostic factors, whereas INHA, INSL4, and PTPRC were considered the unfavorable prognostic factors in LUAD patients. PIK3CG (also termed PI3Kγ) is a protein-coding oncogenic driver gene that encodes a class I phosphoinositide 3-kinase catalytic subunit gamma [39]. PIK3CG activation switches on immune stimulation or suppression in cancer and acute/chronic inflammation. And it could improve the survival of the tumor model in mice when it synergizes with checkpoint inhibitor therapy [40]. The rate of PIK3CG mutations was 0.7% by next-generation sequencing analysis in Swiss never-smoker LUAD patients [41]. Bruton tyrosine kinase (BTK) belongs to the non-receptor tyrosine kinase widely expressed in the hematopoietic lineage cells. Especially, the propagation of BTK signaling is important to B-cell maturation, proliferation, and differentiation [42]. Thus, it is recognized as an attractive drug target for multiple diseases, particularly autoimmune diseases and hematopoietic malignancies related to B lymphocytes [43,44]. It has been recently assumed that BTK might remodel TME to influence the prognosis of LUAD patients based on TCGA data mining [45]. Vascular endothelial growth factor D (VEGFD, alias FIGF) encodes a secreted protein regulating lymphangiogenesis, angiogenesis, and endothelial cell growth as a member of the vascular endothelial growth factor family [46]. The abnormal activity of VEGFD might lead to several types of diseases, such as lymphangioleiomyomatosis, pulmonary diseases, and cardiovascular diseases [47]. A gene signature containing VEGFD plus VEGFA and VEGFB is a prognostic indicator in early-stage non-small cell lung cancer (NSCLC) through detecting multiple gene transcription levels by quantitative polymerase chain reaction [48]. Inhibin subunit alpha (INHA) acts as a member of the TGF-β superfamily. The c.-124 G > A polymorphism of the INHA gene promoter region may cause male infertility in Pakistani [49]. Recent studies have implicated that the function of INHA is linked with androgen-independent metastasis of prostate cancer [50] and angiogenesis of ovarian tumor [51]. However, few reports regarding the relevant role and molecular mechanism of INHA have been described in LUAD. Insulin-like 4 (INSL4), belonging to the insulin superfamily, is a markedly placenta- specific expression [52]. High expression of INSL4 can promote breast cancer invasion and motility by influencing lateral Her2 signaling in vitro cell experiments [53]. Aberrant INSL4 signaling drove the growth and survival of LKB1-deficient lung cancer cells [54]. In an eight-gene-based signature, the expression of INSL4 contributes to 18 some predictive ability for prognostic of LUAD patients [55]. PTPRC (also known as CD45) is a transmembrane protein tyrosine phosphatase and pivotal for T- and B-cell antigen receptor signal transduction [56]. The expression level of PTPRC has been reported to increase in head and neck cancer and has a protective effect for survival in ER-negative breast cancer [57,58]. PTPRC+ cells in the metastatic lymph nodes might be an independent predictor of disease-specific survival of NSCLC patients [59].

Although our signature has been demonstrated that it had the significant predictive potential for LUAD patients’ survival outcome, there are some limitations to this investigation. First, our study was a retrospective analysis of available data from the gene expression profiling in TCGA and GEO. In order to exclude the inherent deficits of retrospective analysis, large-scale prospective research from more centers is needed to evaluate and validate the reliability of the signature. Second, given that only bioinformatics and computational approaches were applied in our study, further molecular biological experiments in vivo and in vitro were required to probe the functional characterization of the six selected genes and elaborate the possible pathogenesis of LUAD. Finally, whether this prognostic signature can predict the survival rate of immunotherapy with other ICBs except for anti-PD1/PDL1 and anti- CTLA4 antibodies, there has still been a great deal of extensive work to be done in the future.

## Conclusion

Taken as a whole, we developed and validated a six-gene signature based on immunologically relevant genes that can reflect not only the immune status of the TME but also stratified LUAD patients into two risk categories, ranking as high-risk and low- risk. In clinical practice, this signature could provide a basis for prognostic information and sensitivity of different therapeutic medications to the LUAD patients to a certain extent. Broadly, our findings might offer the potential molecule biomarkers and facilitate treatment individualization in LUAD patients.

## Data Availability

Any datasets generated and/or analyzed contributing to the conclusions of this article were retrieved from TCGA (https://portal.gdc.cancer.gov/) and GEO (https://www.ncbi.nlm.nih.gov/geo/).

## HIGHLIGHTS

1. A prognostic signature based on immune genes reflecting the TME was established.

2. The RiskScore was related to the abundance of 15 kinds of TIICs.

3. The RiskScore may predict the drug sensitivity.

## ABBREVIATIONS

LUAD: Lung adenocarcinoma; TCGA: The Cancer Genome Atlas; GEO: Gene Expression Omnibus; ImmPort: Immunology Database and Analysis Portal; DEGs: Differentially expressed genes; TME: Tumor microenvironment; ROC: Receiver operating characteristicAUC: Area under the curve; K-M: Kaplan-Meier analysis; TIICs: Tumor-infiltrating immune cells
